# Modeling Alleviative Effects of Ca, Mg, and K on Cu-Induced Oxidative Stress in Grapevine Roots Grown Hydroponically

**DOI:** 10.3390/molecules26175356

**Published:** 2021-09-03

**Authors:** Kai-Wei Juang, Yu-Jin Lo, Bo-Ching Chen

**Affiliations:** 1 Department of Agronomy, National Chiayi University, Chiayi City 600, Taiwan; kwjuang@mail.ncyu.edu.tw; 2 Agricultural Chemistry Division, Taiwan Agricultural Research Institute, Taichung City 413, Taiwan; popo830704@gmail.com; 3 Department of Natural Biotechnology, Nanhua University, Chiayi County 622, Taiwan

**Keywords:** antioxidant, damage level, grapevine root, reactive oxygen species

## Abstract

The aim of this study was to determine the pattern of alleviation effects of calcium (Ca), magnesium (Mg), and potassium (K) on copper (Cu)-induced oxidative toxicity in grapevine roots. Root growth, Cu and cation accumulation, reactive oxygen species (ROS) production, and antioxidant activities were examined in grapevine roots grown in nutrient solutions. The experimental setting was divided into three sets; each set contained a check (Hoagland solution only) and four treatments of simultaneous exposure to 15 μM Cu with four cation levels (i.e., Ca set: 0.5, 2.5, 5, and 10 mM Ca; Mg set: 0.2, 2, 4, and 8 mM Mg; K set: 0.6, 2.4, 4.8, and 9.6 mM K). A damage assessment model (DAM)-based approach was then developed to construct the dose-effect relationship between cation levels and the alleviation effects on Cu-induced oxidative stress. Model parameterization was performed by fitting the model to the experimental data using a nonlinear regression estimation. All data were analyzed by a one-way analysis of variance (ANOVA), followed by multiple comparisons using the least significant difference (LSD) test. The results showed that significant inhibitory effects on the elongation of roots occurred in grapevine roots treated with 15 μM Cu. The addition of Ca and Mg significantly mitigated phytotoxicity in root growth, whereas no significant effect of K treatment on root growth was found. With respect to oxidative stress, ROS and malondialdehyde (MDA) contents, as well as antioxidant (superoxide dismutase (SOD), catalase (CAT), and ascorbate peroxidase (APX)) activities, were stimulated in the roots after exposure to 15 μM Cu for three days. Moreover, H_2_O_2_ levels decreased significantly as Ca, Mg, and K concentrations increased, indicating that the coexistence of these cations effectively alleviated Cu-induced oxidative stress; however, alleviative effects were not observed in the assessment of the MDA content and antioxidant enzyme activities. Based on the DAM, an exponential decay equation was developed and successfully applied to characterize the alleviative effects of Ca, Mg, and K on the H_2_O_2_ content induced by Cu in the roots. In addition, compared with Mg and K, Ca was the most effective cation in the alleviation of Cu-induced ROS. Based on the results, it could be concluded that Cu inhibited root growth and Ca and Mg absorption in grapevines, and stimulated the production of ROS, lipid peroxidation, and antioxidant enzymes. Furthermore, the alleviation effects of cations on Cu-induced ROS were well described by the DAM-based approach developed in the present study.

## 1. Introduction

Copper (Cu), which is an abundant element in the environment, is widely applied in many industries, such as electronics, metallurgy, chemical manufacturing, fertilizers, and fungicides [1]. The background Cu concentration in natural soils ranges from 5 to 30 mg kg^−1^; however, the long-term successive and frequent application of Cu-based fungicides for the control of foliar fungal diseases has led to a significant increase in the Cu concentration in vineyard soils worldwide. An investigation conducted in the wine-growing area of southern Brazil indicated that vineyard soils contained as much as 3200 mg kg^−1^ of Cu [2]. In our previous study in central Taiwan, the highest Cu concentration in the topsoil of vineyards was found to be 100 mg kg^−1^. In Cu-contaminated soils, grapevines may accumulate large amounts of Cu in their roots, aboveground parts, and even their fruit.

Copper has a dual effect on plants [1]. At an optimal level, Cu is an important micronutrient in regulatory metabolic processes, including photosynthesis and carbohydrate partitioning in plants [3,4,5]. However, excess Cu in vineyard soils may result in several phytotoxic effects on grapevines, such as cell membrane damage, nutritional imbalances, leaf chlorosis, growth inhibition, a reduction in chlorophyll content, and even death [6,7]. Consequently, a high concentration of Cu in vineyard soils leads to a decline in grape productivity and economic losses in the grape industry. Furthermore, many previous studies have indicated that the exposure of the general population to Cu is primarily through food chain transfer, which indicates the importance of assessing potential human health risks in the consumption of grapes and grape products [8,9]. Therefore, it is necessary to study Cu accumulation in grapevines and its implications for the assessment of human health risks in food chain transfer.

Traditionally, metal concentrations in plants’ organs, as well as the subsequent inhibition effects of biomass and elongation, have widely been considered the endpoints of chronic phytotoxicity in terrestrial ecosystems. Several studies have been conducted to determine the concentration–growth inhibition relationship in grapevines exposed to excess Cu [6,10,11]. In addition to physiological responses, it is recognized that heavy metals may cause molecular damage to plants through reactive oxygen species (ROS) formation. The overproduction of ROS is then regulated by the antioxidative system, which consists of protective enzymes (e.g., superoxide dismutase (SOD) and catalase (CAT)) and low-molecular-weight antioxidants (e.g., glutathione and phenolic compounds). Several studies have indicated that the presence of Cu in plants above threshold concentrations induces membrane lipid peroxidation and ion leakage from cells, in addition to oxidative damage to proteins and nucleic acids [2,12,13,14]. Recently, some studies have utilized changes in the activities of antioxidative enzymes and the content of soluble antioxidants as biomarkers of the Cu toxicity of grapevines [11,15,16,17].

It has been recognized that the presence of coexisting cations and anions may influence the phytoavailability of metals in soil environments [17,18,19]; therefore, to accurately assess the toxic effects of metals on terrestrial organisms, some physicochemical and empirical models have been developed to characterize the effects of coexisting elements on metal toxicity. The most popular approaches to predicting the interactive effects of metal toxicity include the free ion activity model (FIAM), the biotic ligand model (BLM), and the electrostatic toxicity model (ETM) [19]. In our previously published work, for example, an FIAM-based concept was successfully applied to model the effects of magnesium (Mg) on Cu toxicity in the root elongation of grapevine [18]. In addition to the above physicochemical models, the damage assessment model (DAM), which is based on the level of biological damage at the target site, was developed by Lee et al. [20] as a reliable tool for the toxicity assessment of chemicals. In the last two decades, the concept of DAM has been widely and successfully applied to assess aquatic toxicity caused by heavy metals [21,22,23]. More recently, Gao et al. [24] used the DAM to study the oxidative stress-dependent toxicity of Cd and Pb in zebrafish. To the authors’ knowledge, no previous study has employed DAM to assess the influence of competitive cations on the oxidative effects of heavy metals on terrestrial plants such as grapevines.

Therefore, in the present study, a hydroponic experiment was conducted to examine the toxic effects of Cu on grapevines under various cations (Ca, Mg, and K) in treatment combinations. The accumulation and translocation of Cu and cations in grapevine roots were then determined. The ROS (H_2_O_2_) and malondialdehyde (MDA) contents, as well as the antioxidant enzyme (SOD, CAT, and ascorbate peroxidase (APX)) levels in the roots, were investigated under different exposures. Based on the concept of DAM, special attention was paid to determining the relationship of the cation levels to the mitigation of oxidative stress caused by Cu.

## 2. Results

### 2.1. Effects of Cation Treatments on Rroot Growth

**Figure 1:**
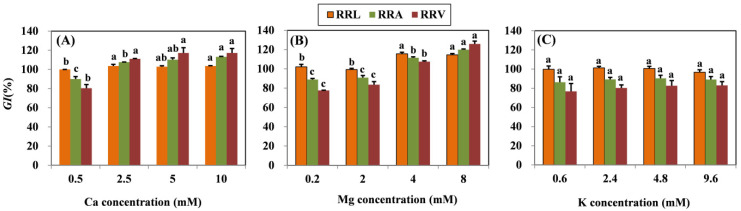
Root growth response index (*GI*) (*RRL*, *RRA*, and *RRV*) of grapevine seedlings exposed to 15 μM Cu for three days under various (**A**) Ca, (**B**) Mg, and (**C**) K treatments. Each value represents the mean of three replicates ± SE (standard error). Within each growth response, error bars with the same lowercase letters indicate that the mean values are not significantly different at *p* < 0.05 among various cation treatments (LSD test).

According to the ANOVA, the treatment effects of different Ca, Mg, and K levels with coexistent 15 μM Cu on *RRL*, *RRA*, and *RRV* were significant. Results showed that all root growth indexes (*RRL*, *RRA*, and *RRV*) increased significantly in grapevine seedlings subjected to Mg concentrations higher than 4 mM (Figure 1). In the Ca amendment, growth indexes also increased as concentrations of Ca increased, except the *RRL* of the 5 mM Ca treatment. The highest Ca concentration (10 mM) resulted in the increase in *RRL*, *RRA*, and *RRV* by 3.62, 23.19, and 36.71%, respectively. With the highest Mg concentration (8 mM), the *RRL*, *RRA*, and *RRV* increased by 12.31, 30.95, and 48.28%, respectively. No significant difference in the root growth index was observed in the K treatment.

### 2.2. Cu and Cation Accumulation

**Figure 2:**
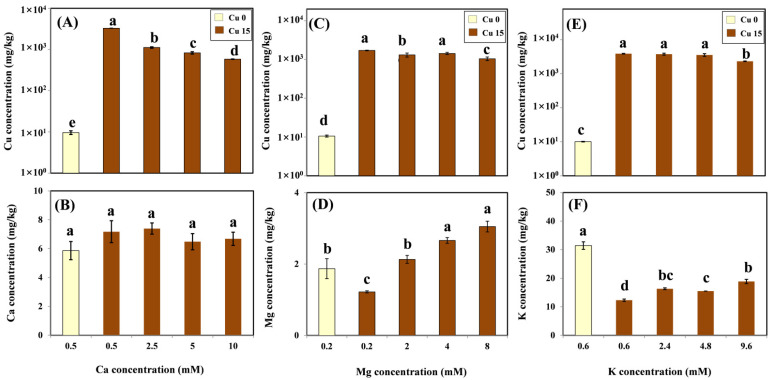
Cu concentration (log scale) and cation concentration in roots of grapevine seedlings after three-day exposure to various Ca (**A**,**B**), Mg (**C**,**D**), and K (**E**,**F**) treatments with or without 15 μM Cu treatment. Each value represents the mean of three replicates ± SE (standard error). Error bars with the same lowercase letters indicate that the mean values were not significantly different at *p* < 0.05 among various cation treatments (LSD test).

According to the ANOVA, the treatment effects of different Ca, Mg, and K levels with coexistent 15 μM Cu on Cu and cation concentrations in roots were significant. Cu and cation accumulation in the root in relation to cation concentrations with and without Cu treatment was tested by LSD and shown in Figure 2. The mean background Cu concentration ranged from 9.64 to 10.58 mg/kg. After exposure to 15 μM Cu for three days, as expected, the mean Cu concentration significantly increased from 1709 to 3884 mg/kg in the root. In the presence of Ca in the Cu treatment medium, there was a significant decrease in Cu concentration in roots under an increasing Ca exposure (Figure 2A). However, no significant differences were observed in Ca levels in roots exposed to various Ca concentrations, with or without Cu treatment. Ca concentrations in the roots were not affected by the treatments and were maintained at steady levels ranging from 5.86 to 7.39 mg/kg (Figure 2B). With Mg present in the Cu treatment medium, Cu concentrations in the roots decreased slightly from 1709 to 1045 mg/kg (Figure 2C). The amendment of Mg significantly enhanced Mg accumulation in the roots, reaching 3.05 mg/kg at the highest Mg exposure level (Figure 2D). Except for the highest K treatment (9.6 mM), the K amendment did not affect the root’s Cu accumulation (Figure 2E). The K concentration in the roots decreased by 61% on average with the addition of 15 μM Cu compared with the treatment without the addition of Cu. Nevertheless, the concentration of this nutrient in the root significantly increased with increasing K concentrations in the solution (Figure 2F).

In order to further characterize the effects of cations on Cu accumulation in the roots, the bioconcentration factor (*BCF*) of Cu was calculated; the results are shown in Table 1. The *BCF* decreased linearly as Ca concentrations increased. The Mg treatment showed an increase in *BCF* of up to 4 mM Mg; however, *BCF* decreased with the addition of the highest dose (8 mM). In the K treatment, no significant difference in *BCF* was observed with the additions of 2.4 and 4.8 mM K, whereas the lowest *BCF* was observed at the highest dose (9.6 mM).

### 2.3. Cu-Induced Oxidative Stress and Damage Mitigation Effects of Coexisting Cations

**Figure 3:**
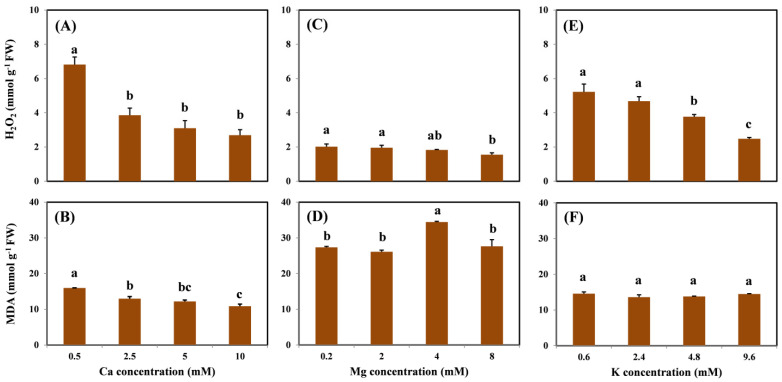
H_2_O_2_ and MDA concentrations in the roots of grapevine seedlings after three-day exposure to 15 μM Cu under various Ca (**A**,**B**), Mg (**C**,**D**), and K (**E**,**F**) treatments. Each value represents the mean of three replicates ± SE (standard error). Error bars with the same lowercase letters indicate that mean values are not significantly different at *p* < 0.05 among various cation treatments (LSD test).

The profiles of H_2_O_2_ and MDA concentrations in the roots are shown in Figure 3. There was a significantly decreasing trend in root H_2_O_2_ concentration as Ca, Mg, and K levels increased (Figure 3A,C,E). Compared with the treatment without coexisting cations, H_2_O_2_ concentrations decreased by 61%, 23%, and 52% on average with the highest Ca, Mg, and K treatment levels, respectively. Moreover, H_2_O_2_ concentrations were maintained at a steady level from 1.55 to 2.69 mmol/g in the highest cation treatment. Only the addition of Ca showed a similar trend in the MDA concentration, which decreased by 32% on average with the addition of 10 mM Ca compared with the treatment without Ca (Figure 3B). No dose–effect relationship was observed in MDA under various Mg and K treatments (Figure 3D,F).

**Figure 4:**
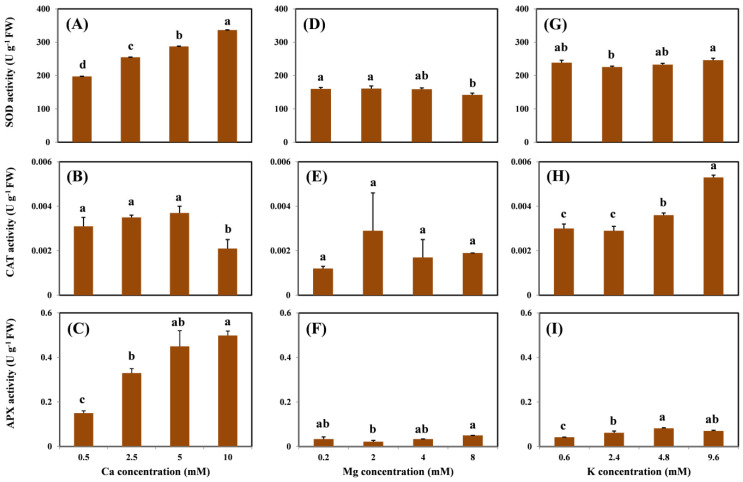
SOD, CAT, and APX activity in the roots of grapevine seedlings after three-day exposure to 15 μM Cu under various Ca (**A**–**C**), Mg (**D**–**F**), and K (**G**–**I**) treatments. Each value represents the mean of three replicates ± SE (standard error). Error bars with the same lowercase letters indicate that the mean values are not significantly different at *p* < 0.05 among various cation treatments (LSD test).

SOD activity in the roots increased with increasing Ca doses in the exposure medium (Figure 4A). However, the addition of Mg and K did not change the root SOD activity, except for the highest Mg treatment (Figure 4D,G). In contrast, a different trend of CAT activity in the root was observed under treatment with different cations. The highest Ca concentration (i.e., 10 mM) decreased the CAT activity compared with the treatment without Ca (Figure 4B); on the contrary, the addition of K significantly increased the root CAT activity (Figure 4H). No statistical differences were observed between the effects of the increased doses of Mg (Figure 4E). However, Ca and K levels significantly increased the activity of APX in the roots (Figure 4C,I). Similar to the CAT activity, no significant difference in the APX activity was observed with increased doses of Mg (Figure 4F).

In this study, the dose–effect relationship between H_2_O_2_ and MDA concentrations in the roots and the cation concentration in the medium was expressed by a DAM-based conceptual model (Equation (4))—an exponential decay model that can be employed to better characterize the alleviation effects of cations on oxidative stress caused by Cu. The nonlinear regression results obtained from the best fit of Equation (4) to the experimental data are shown in Table 2. The results showed that the dose alleviation effects of cations on H_2_O_2_ stress were well described by the model, whereas the *R*^2^ values ranged from 0.809 to 0.975 (*p* < 0.05). However, regarding MDA, the model was only capable of describing the alleviation effect of Ca at a relatively lower *R*^2^ value of 0.830. *DL*_0_ (i.e., damage level without a coexisting cation) is shown in Table 2. In H_2_O_2_, the *DL*_0_ values ranged from 2.02 to 6.82, indicating that the level of H_2_O_2_ was approximately three- to eight-fold under Cu treatment compared with the control. The level of damage mitigation by the cation on Cu oxidative stress to the root, *h*, is a critical parameter in the model, as shown in Table 2. With respect to H_2_O_2_, the *h* value was highest in the Ca treatment (0.139 mM^−1^), whereas the lowest *h* value was observed in the Mg treatment (0.03 mM^−1^).

## 3. Discussion

It is generally recognized that plant roots are the most sensitive targets of environmental stress, such as an excess exposure to metals. Therefore, changes in the root growth and morphology have often been used as a bioindicator of metal phytotoxicity [17,25]. In the present study, some morphological damage, such as the darkening and thickening of the root cap and less root hair formation, was observed after treatment with 15 μM of Cu for three days (figure not shown). These symptoms may result in the lower absorption of essential nutrients, thus resulting in reduced root growth. The phytotoxicity symptoms of Cu on vine roots have been widely described in the literature [10,16,17,26,27]. Ambrosini et al. [26] indicated that excess Cu may shorten the cell differentiation region and increase the diameter at the root apex of young vines, thus inhibiting root growth. Tiecher et al. [16] further proposed that root thickening is a defense strategy of grapevines in response to Cu stress. More recently, Castro et al. [27] studied the phytotoxic effects of excess Cu on the roots of four grapevine varieties and concluded that a reduction in root elongation occurred between 10 and 50 μM of Cu exposure, which is consistent with the present results.

Previous studies adopted strategies for the reduction of the toxic effects of Cu on plants. Ferraire et al. [5] showed that the inoculation of arbuscular mycorrhizal fungi (AMF) and the application of phosphate-related fertilizer ameliorated the Cu toxicity in *Mucuna cinereum* by decreasing the availability of Cu^2+^ in the soil solution. Regarding Cu toxicity in grapevines, previous studies showed that some amendments, such as limestone, vermicompost, and calcium silicate, were effective in reducing the Cu phytotoxicity in young vines, which was due to increasing pH in the soil, leading to a decreased Cu^2+^ availability [14,26,28]. Essential macronutrients, such as Ca, Mg, and K, are important in plant growth and development. In the present study, it was observed that some new lateral hair occurred at the root apex with the addition of the highest treatment concentrations of Mg and Ca. Moreover, all of the root growth indexes increased as the Ca and Mg concentrations increased, indicating that the addition of these two cations is a promising strategy for the alleviation of Cu toxicity in vine roots. These results are consistent with our previously published studies, which revealed that Cu toxicity symptoms are significantly alleviated by Mg and Ca [18,29,30]. The alleviation effects may be due to the prevention of Cu from binding to toxic action sites at plasma membrane surfaces by Mg and Ca. In addition, these two elements are important for the constitution of the pectin in the middle lamella because they strengthen cell walls and reduce the toxic effects of Cu on root tissue [26].

In the present study, after exposure to 15 μM Cu for three days, the Cu concentration reached values between 1709 and 3884 mg/kg in the root, which were higher than those considered normal in vine roots, and, thus, could be toxic in grapevines. This result is in line with previous studies showing that the root is the main organ of Cu accumulation in grapevines [6,10,16,29]. The high capacity of Cu accumulation in roots is generally regarded as a defense mechanism of grapevines that experience Cu stress. Furthermore, Ambrosini et al.’s results indicated that the highest proportion of Cu accumulated in grapevine roots was located in the apoplast rather than in the symplast, thus restricting its translocation to shoots and reducing its phytotoxicity [4]. However, the coexistence of Ca and Mg led to a significant decrease in the Cu concentration of vine roots in the present study, implying that Ca and Mg compete for sorption at the plasma membrane surface of grapevine roots. This result is in line with Ambrosini et al.’s findings, which indicated that elements with the same valence, such as Ca, Mg, and Cu, may compete for similar binding sites on the root surfaces of grapevines [26]. In the present study, unlike Ca and Mg, a significant decrease in the Cu level was observed only in the highest K treatment (9.6 mM). This result indicated that a high level of K had an inhibitory effect on Cu activity in the solution, thus restricting the absorption of Cu by the root surface.

Macronutrients, such as Ca, Mg, and K, are important in plant growth and development. In the present study, the addition of 15 μM Cu significantly reduced Mg and K levels in the grapevine root (Figure 2D,F), which may result in an imbalance of nutritional status, and thus, affect the normal growth of the grapevine. This result is in line with Mateos-Naranjo et al.’s study on the grazing species *Atriplex halimus* [31], as well as Ambrosini et al.’s and Juang et al.’s studies on grapevines [4,18]. However, the amendments of Mg and K in the solution significantly increased the levels of these two macronutrients in the roots in the present study. It is generally recognized that Mg plays a major role in the synthesis of chlorophyll, whereas K is important in maintaining cell turgor, stomatal activity, membrane potential, and mobilizing assimilates [26,32]. Therefore, the enrichment of these two nutrients may be helpful in strengthening plants and reducing the toxic effects of Cu on the root tissue. In contrast, the concentration of Ca content in the roots was not significantly affected by the addition of Cu and/or Ca. This result is consistent with that of Ambrosini et al. [4], who worked with “Red Niagara” plantlets and also found a stable Ca accumulation in roots exposed to various Cu levels from 25 to 175 mg/kg. It is most likely that Ca may reduce the toxic effects of Cu by incorporating it into Ca-oxalate crystals in grapevines [26]. Consequently, the Ca level in its roots may be regulated by the grapevine and maintained at normal levels, even under Cu stress.

Under Cu stress, the concentration of ROS increases in plants [1]. Recently, excess Cu exposure has been reported to stimulate the production of ROS, such as H_2_O_2_ in grapevine roots, potentially increasing oxygen-induced cellular damage [7,17]. The production of H_2_O_2_ could then initiate the process of lipid peroxidation, resulting in the generation of MDA. Therefore, the levels of H_2_O_2_ and MDA are generally regarded as biomarkers of ROS-induced cellular injury [1,7,12,17]. The results of the present study showed that the concentrations of H_2_O_2_ and MDA in the grapevine roots were significantly enhanced by the Cu treatment, indicating that oxidative stress was induced by Cu. A similar result was observed by Zhou et al. [7], who proposed that the addition of 120 μM Cu significantly increased the contents of H_2_O_2_ and MDA in grapevine roots. However, all levels of H_2_O_2_ significantly decreased as Ca, Mg, and K concentrations were increased in the solution. This result further indicates that cation treatment could alleviate Cu-induced oxidative stress by decreasing the degree of ROS in the roots. Unlike H_2_O_2_, the MDA content was affected only by the addition of Ca. This result suggests that the content of H_2_O_2_ is a more direct and appropriate biomarker of Cu-induced oxidative stress. In addition, compared with Mg and K, Ca is more effective for the alleviation of oxidative toxicity, which may be attributed to the superior performance of Ca in the reduction of Cu content in the roots. Although the effects of macronutrients on Cu-induced toxicity have been investigated [4,6,29,30,31], no previous study has characterized the alleviation effects of cations on Cu-induced oxidative stress.

SOD, CAT, and APX are important antioxidant enzymes involved in the removal of ROS. Thus, an increase in ROS is related to an increase in the activity of the antioxidant enzymes. However, in this study, the antioxidant enzyme activity varied and was not well correlated with the concentration of H_2_O_2_ in the roots. Indeed, the inactivation or activation of these antioxidant enzymes depends not only on H_2_O_2_ levels but also on plant species. Shabbir et al. [1] indicated that, instead of the activation of antioxidant enzymes, genes and transporter proteins inside plant tissues may also regulate Cu-induced ROS production.

In ecotoxicology, it is critical to construct the dose–response profile of an organism exposed to xenobiotics. In the present study, a DAM-concept-based exponential decay model was successfully employed to determine the relationship between dose alleviation effects of cation concentrations in the solution and Cu-induced ROS content in grapevine roots. However, the model failed to show the effects of coexisting cations on the activity of antioxidant enzymes. This result indicates that the H_2_O_2_ molecule is a key signaling molecule associated with metabolic pathways in grapevines under Cu stress [1]. Moreover, it was previously reported that H_2_O_2_ may be removed by other enzymes, such as guaiacol peroxidase (GPX). Thus, no or very little effect was found regarding the elimination of H_2_O_2_ by SOD, CAT, and APX [12]. A similar result in spinach seedlings was obtained by Gong et al. [19]. Therefore, further studies are recommended to characterize the relationship between the ROS content and activity of ROS-removing enzymes. In this study, the slope of the dose–response profile became steeper as the h value increased (i.e., the level of damage mitigation by the cation on Cu oxidative stress in the root), representing the better mitigation effect of the cation on oxidative damage. Based on our results, the addition of Ca is most effective in the alleviation of H_2_O_2_ stress caused by Cu, followed by K and then Mg. To the authors’ knowledge, this study is the first to apply the DAM to assess the effects of competitive cations on the oxidative effects of Cu on grapevines. The methodology developed in this study could be applied in future assessments and comparisons of the alleviation effects of coexisting cations on heavy-metal-induced oxidative stress.

## 4. Materials and Methods

### 4.1. Plant Material and Hydroponic Experiment

The annual shoots of Kyoho grapevine (*Vitis vinifera* L.) were collected from vine-growing areas in central Taiwan and transferred to the laboratory. Each shoot was divided into several cuttings so that each cutting contained two nodes and three dormant buds. One end of each grapevine cutting was placed in distilled water for 30 days until enough leaves (three or four) completely emerged, and the axillary buds were then utilized as tissue culture. After the leaves were removed, the axillary buds were sterilized, washed, and transferred to a test tube containing 15 mL MS basal medium for another 30 days. The node of the seedling was inoculated on a 15 mL 1/2 MS medium for the subculture. Microshoots were rooted on media supplemented by 0.2 mg kg^−1^ indole-3-butyric acid (IBA). When the axillary bud explants rooted and shoots had emerged in vivo, healthy tissue culture seedlings were selected and transplanted into a peat moss mixture (Plantaflor^®^ Blocking Substrate, Plantaflor Humus Verkaufs-GmbH, Vechta, Germany) and acclimated in vitro. After 45 days of acclimation, seedlings with three new leaves were used in the hydroponic experiment. A grapevine root was pruned to 5 cm and transplanted into a 0.7 L polypropylene bottle filled with 10% modified Hoagland solution (0.5 mM KCl, 0.5 mM CaCl_2_, 0.1 mM KH_2_PO_4_, 0.2 mM MgSO_4_, 0.01 mM Fe-EDTA, and 1.5 mM NH_4_NO_3_) for two days. 

The experimental design consisted of a completely randomized design (CRD) where the experimental units were randomly assigned to a series of treatment levels. Calcium chloride (CaCl_2_·H_2_O) was applied to the solution to provide one set of four test solutions of 0.5, 2.5, 5, and 10 mM Ca (the Ca set) with a coexistent Cu level of 15 μM, and a check (CK) was set at 0.5 mM Ca without Cu addition. Magnesium sulfate (MgSO_4_·7H_2_O) was applied to the solution to provide another set of four test solutions of 0.2, 2, 4, and 8 mM Mg (the Mg set) with a coexistent Cu level of 15 μM, and a check (CK) was set at 0.2 mM Mg without Cu addition. Potassium chloride (KCl) was applied to the solution to provide a final set of four test solutions of 0.6, 2.4, 4.8, and 9.6 mM K (the K set) with a coexistent Cu level of 15 μM, and a check (CK) was set at 0.6 mM K without Cu addition. In each set, there were five treatment levels including CK. Copper sulfate (CuSO_4_) was added to the test solution to prepare Cu-spiked solutions. One seedling was placed in one bottle containing the treatment solution. Each experimental unit comprised three bottles as replicates. The test solution was renewed every day. The experiment was conducted in growth chambers with a fixed temperature (25 ± 0.5 °C during the day and 23 ± 0.5 °C at night) and relative humidity (75%). The light cycle was 16:8 (light:dark). Test media were aerated throughout the experiment. After exposure for three days, the seedlings in each bottle were harvested and thoroughly washed with deionized water before analysis.

### 4.2. Root Growth

To assess the effects of Cu and coexisting cations on the growth of grapevine roots, the roots of each exposed group were photographed before and after the experiment and then transferred into draft files. The total root length (*tRL*), total root surface area (*tRA*), and total root volume (*tRV*) were then calculated using DIGIROOT V. 2.5 software [33]. The relative root length (*RRL*), relative root surface area (*RRA*), and relative root volume (*RRV*) of each exposed group, with respect to the control, was estimated as follows:

(1) $$GI=\frac{\Delta G}{G_{0}}\times 100\%$$

where *GI* (%) is the growth response index of the vine root (*RRL*, *RRA*, and *RRV*), *G*_0_ is the growth parameter (*tRL*, *tRA*, and *tRV*) before the exposure experiment (i.e., day 0), and Δ*G* represents the difference in growth parameter after (i.e., day 3) and before the experiment.

### 4.3. Chemical Analysis

The roots and leaves of the grapevine seedlings were separately harvested using clean vinyl gloves and then placed in clean polyethylene bags. These samples were oven dried at 180 °C for four to six hours, and the dry weights of plant tissues were recorded as 0.1 g. The plant samples were ground and digested in HNO_3_/HClO_4_ (4:1 *v*/*v*), and Ca, Mg, and Cu concentrations in both the plants and the hydroponic medium were then determined using a flame atomic absorption spectrophotometer (iCE 3000 Series, Thermo Scientific, Waltham, MA, USA). Potassium concentration was determined by inductively coupled plasma mass spectrometry (ICP-MS). All chemical analyses were performed in duplicate.

### 4.4. Bioconcentration Factor

In the present study, to investigate the absorption and accumulation of Cu in grapevine roots under various cation treatments, a bioconcentration factor (*BCF*) was introduced and calculated as follows:

(2) $$BCF=\frac{Cu_{root}}{\left\{Cu^{2+}\right\}}$$

where *Cu_root_* is the Cu concentration in grapevine root (mg/kg) and {Cu^2+^} represents free Cu activity (μM). Cu activity was calculated according to Skoog et al.’s [34] method using Visual MINTEQ software.

### 4.5. Determination of ROS Content and Antioxidative Enzymes

In order to extract antioxidant enzymes (SOD, CAT, and APX) and H_2_O_2_, 0.1 g of grapevine root and leaf sample in the hydroponic experiment were homogenized with 5 mL of 100 mM potassium phosphate buffer (PPB) (pH 7.5) and 0.1 g of polyvinylpolypyrrolidone (PVPP) in a prechilled mortar and pestle. The homogenate was centrifuged at 12,000× *g* for 20 min at 4 °C to collect the supernatant for estimation of antioxidant enzymes and H_2_O_2_.

SOD activity was assayed according to Beauchamp and Fridovich [35]. The assay mixture consisted of a total volume of 0.75 mL, which contained 0.1 M potassium phosphate, pH 7.8, 1 mM Na_2_-EDTA, 130 mM methionine, 0.63 mM nitro blue tetrazolium (NBT), 7.5 μM riboflavin, and the sample. Illumination of the reaction mixtures caused the formation of blue formazan and increased absorbance at 560 nm. One unit of SOD was defined as the amount of enzyme that inhibited the reduction of NBT by 50% at 560 nm.

CAT activity was determined according to Kato and Shimizu [36]. A total of 0.3 mL of enzyme extract was added to 2 mL of the reaction mixture containing 1 mL of 100 mM phosphate buffer (pH 7.0) and 0.05 mL of 10 mM H_2_O_2_. The reaction was started by adding the enzyme extract, after which the absorbance was recorded continuously every 10 s. Eventually, the decrease in absorbance at 240 nm for 1 min was measured to express CAT activity in the plant tissue. Absorbance values at 240 nm were recorded using a UV-visible spectrophotometer (Evolution 60S UV-Visible, Thermo Scientific). The activity was calculated using the extinction coefficient (40 mM^−1^ cm^−1^) of H_2_O_2_.

APX activity was measured by the reduction of ascorbic acid in terms of the absorbance at 290 nm, with an extinction coefficient of 2.8 mM^−1^ cm^−1^, according to Nakano and Asada [37]. A total of 0.15 mL of enzyme extract was added to 2 mL of the reaction mixture containing 0.5 mL of 50 mM phosphate buffer (pH 7.0), 0.2 mL of 1-mM EDTA-Na_2_, 0.5 mL of 0.1-mM ascorbate, and 0.1 mL of 10-mM H_2_O_2_. The reaction was initiated by adding the enzyme extract, after which the absorbance was recorded continuously every 10 s. Eventually, the decrease in absorbance at 290 nm for 1 min was measured to express the APX activity in the plant tissue. The absorbance values at 290 nm were recorded using a UV-visible spectrophotometer (Evolution 60S UV-Visible, Thermo Scientific).

The H_2_O_2_ content of the grapevine seedlings was determined according to Jana and Choudhuri [38]. The reaction mixture contained 0.1 mL of 0.1% (*w*/*v*) titanium chloride dissolved in 20% (*v*/*v*) H_2_SO_4_ and supernatant at a proportion of 1:2 at 1000× *g* for 10 min in order to achieve homogeneity. The content was evaluated by comparing its absorbance at 410 nm with a standard calibration curve.

The level of lipid peroxidation was estimated following Heath and Packer’s method [39], in which the concentration of malondialdehyde (MDA) was measured as a product of lipid peroxidation by reaction with thiobarbituric acid (TBA). Root and leaf samples were homogenized with 2 mL of 5% (*w*/*v*) trichloroacetic acid (TCA) in a prechilled mortar and pestle and centrifuged at 10,000× *g* for 10 min at 4 °C. Then, 1 mL of the supernatant and 4 mL of 20% TCA containing 0.5% TBA were added. The mixture was heated at 95 °C for 30 min and quickly cooled in an ice bath for 15 min. After centrifugation at 3000× *g* for 10 min, the absorbance of the supernatant at 532 nm was recorded and corrected by measurement at 450 nm and 600 nm.

### 4.6. Damage Mitigation Model

In the present study, the oxidative-stress-based damage model developed by Gao et al. [24] was employed to model the phytotoxicity of Cu in grapevines. The damage level (*DL*) represented the change in ROS or antioxidant enzyme compared with the control group, which was estimated as follows:

(3) $$DL=\left(\frac{D_{t}}{D_{c}}-1\right)$$

where *D_t_* is the ROS concentration or antioxidant enzyme activity in the treatment group and *D_c_* is that in the control group. According to previously published works, metal toxicity may be affected by coexisting cations, such as Ca, Mg, and K [26,29,30]. Therefore, in this study, an exponential decay model was applied to represent the mitigation effects of cations on Cu oxidative stress in grapevine root:

(4) $$DL\left(C_{x}\right)=DL_{0}\cdot e^{-h_{x}\cdot C_{x}}$$

where *C_x_* is the concentration of cation *x* (mM), *DL*_0_ represents the damage level without coexisting cations, and *h_x_* denotes the level of damage mitigation of cation *x* on Cu oxidative stress to grapevine root (mM^−1^). Therefore, for each cation set, *h_x_* was determined by fitting Equation (4) to the experimental data using a nonlinear regression estimation. In Equation (4), the inherent assumption is that the damage level decreases as the coexisting cation concentration increases.

### 4.7. Statistical Analysis

Statistical analyses of the root elongation, as well as Cu and cation (Ca, Mg, and K) concentrations in plant samples and in treated solutions, were conducted. According to the CRD, the data were analyzed by a one-way analysis of variance (ANOVA) followed by mutually multiple comparisons for any two treatment levels using the least significant difference (LSD) test. The level of significance is set at *p* < 0.05. Curve fittings for damage mitigation modeling were performed using a nonlinear regression. All statistical analyses were performed using Statistica software (StatSoft, Tulsa, OK, USA).

## Figures and Tables

**Table 1:** Bioconcentration factor (*BCF*) of {Cu^2+^} activity from solution added to grapevine root after exposure to 15 μM Cu for three days under various Ca, Mg, and K treatments.

Coexisting Cation	Cation Concentration (mM)	*BCF* *
Ca	0.5	299a
	2.5	124b
	5	95.3c
	10	76.2c
Mg	0.2	155b
	2	177ab
	4	206a
	8	165b
K	0.6	353ab
	2.4	353a
	4.8	368a
	9.6	260b

* Each value represents the means of three replicates. Means followed by the same lowercase letter did not differ between cation concentrations in the same cation treatment by the LSD test (*p* < 0.05).

**Table 2:** Optimal fit of Equation (4) to the data obtained from a three-day experiment in which a grapevine was exposed to Cu and coexisting cations.

Coexisting Cation	Oxidative Parameter	*DL*_0_	*h* (mM^−1^)	*p* Value	*R* ^2^
Ca	H_2_O_2_	6.82	0.139 ± 0.030	0.019	0.809
	MDA	15.97	0.0457 ± 0.007	0.008	0.830
Mg	H_2_O_2_	2.02	0.03 ± 0.003	0.002	0.952
	MDA	-	-	ns *	-
K	H_2_O_2_	5.22	0.071 ± 0.006	0.001	0.975
	MDA	-	-	ns	-

* ns: not significant at *p* < 0.05.

## Data Availability

The data presented in this study are available on request from the corresponding author. The data are not publicly available due to the sizeable raw data files requiring storage on an offline hard drive for safekeeping.

## Sample Availability

Samples are not available due to being perishable goods.
